# Increasingly severe malnutrition according to the geriatric nutritional risk index is associated with a greater risk of postoperative adverse events

**DOI:** 10.1007/s00590-025-04317-1

**Published:** 2025-05-23

**Authors:** Steven H. Liu, Brandon Lung, Jane Burgan, Rachel A. Loyst, Rebecca Liu, Allen Bramian, James J. Nicholson, Russell N. Stitzlein

**Affiliations:** 1 University of Southern California, Los Angeles, USA; 2 University of California, Irvine Medical Center, Orange, USA; 3 Stony Brook Medicine, Stony Brook, USA

**Keywords:** Revision total knee arthroplasty, Malnutrition, Geriatric, Geriatric nutritional risk index, Complications

## Abstract

**Background:**

This study investigates the association between the geriatric nutritional risk index (GNRI), a readily available index measuring the risk of malnutrition, and 30-day postoperative complications following revision total knee arthroplasty (rTKA).

**Methods:**

The American College of Surgeons National Surgical Quality Improvement Program database was queried for all patients ≥ 65 who underwent rTKA between 2015 and 2021. The study population was divided into three groups based on preoperative GNRI: normal/reference (GNRI > 98), moderate malnutrition (92 ≤ GNRI ≤ 98), and severe malnutrition (GNRI < 92). Multivariate logistic regression analysis was conducted to investigate the association between preoperative GNRI and postoperative complications.

**Results:**

Compared to normal nutrition, moderate malnutrition was independently significantly associated with a greater likelihood of experiencing any complication, blood transfusions, surgical site infection (SSI), non-home discharge, readmission, length of stay (LOS) > 2 days, and mortality. Severe malnutrition was independently significantly associated with a greater likelihood of experiencing any complication, septic shock, pneumonia, unplanned reintubation, cardiac arrest or myocardial infarction, stroke, blood transfusions, still on ventilator > 48 h, SSI, wound dehiscence, acute renal failure, non-home discharge, readmission, unplanned reoperation, LOS > 2 days, and mortality. Severe malnutrition was independently significantly associated with a greater number of complications and had a stronger association with complications compared to moderate malnutrition.

**Conclusion:**

Malnutrition identified by GNRI has strong predictive value for short-term postoperative complications following rTKA in geriatric patients and may have utility as an adjunctive risk stratification tool for geriatric patients undergoing rTKA.

## Introduction

Total knee arthroplasty (TKA) is an effective surgical treatment option for patients with debilitating knee osteoarthritis (OA) seeking to alleviate pain and improve quality of life [1, 2]. Osteoarthritis is the most prevalent joint disease in the USA, with knee OA accounting for 80% of the disease’s total burden [3]. The geriatric population is rapidly growing in the USA, resulting in more adults suffering from OA and thus a greater prevalence of TKA [4, 5]. Revision total knee arthroplasty (rTKA) procedures, which are often performed to correct complications following primary TKA, such as prosthetic infections and joint loosening, are also increasing as more TKAs are being performed [6]. The incidence of both TKA and rTKA is expected to rise, with a projected 90% annual increase in rTKA compared to a 43% annual increase in TKA [7].

With a rising incidence of rTKAs, it has become increasingly pertinent to understand patient risk factors and conditions associated with poorer postoperative outcomes. Malnutrition is a well-documented risk factor in orthopedic joint surgery that has been linked to an increased rate of adverse postoperative outcomes, including infection, myocardial infarction, increased length of hospital stay, readmission, and return visits to the emergency department [8–11].

In the past, serum albumin has been used as a marker for malnutrition. However, due to mixed evidence regarding its validity as a proxy for malnutrition, newer risk indices such as the geriatric nutritional risk index (GNRI) have been developed [12]. GNRI, which assesses the risk of malnutrition in geriatric patients, is calculated using serum albumin and ideal body weight [13]. Previous literature has demonstrated that GNRI is a predictor of adverse postoperative outcomes following total joint arthroplasty (TJA) [14]. However, its utility in assessing the prognosis of geriatric patients who undergo rTKA has not been studied.

The purpose of this study is to investigate the relationship between GNRI and postoperative outcomes following rTKA. We hypothesized that there is an increased risk of early postoperative complications in patients with GNRI indicative of malnutrition compared to patients with normal GNRI.

## Materials and methods

We queried the American College of Surgeons National Surgical Quality Improvement Program (ACS-NSQIP) database for all patients who underwent rTKA between 2015 and 2021. This study was exempt from approval by our University’s Institutional Review Board because the NSQIP database is fully de-identified. Data in the NSQIP database are obtained from over 600 hospitals in the USA, are collected by trained Surgical Clinical Reviewers, and provide validates 30-day surgical outcomes.

The *Current Procedural Terminology* (CPT) codes corresponding to rTKA (27,486—one component and 27,487—femoral and entire tibial component) were used to identify 30,557 patients who underwent rTKA between 2015 and 2021. NSQIP inherently excludes all cases of patients under 18 years of age and all cases with primary admission criteria related to trauma. Next, 13,038 patients with missing height, weight, or preoperative albumin values required to calculate GNRI were excluded, leaving 17,519 patients. Next, 7,814 cases were excluded for missing American Society of Anesthesiologists (ASA) classification, unknown discharges destination, functional health status, or age < 65. Next, revisions secondary to periprosthetic joint infections were excluded because the NSQIP does not provide information related to the chronicity of the infection (acute vs. chronic) or the type of revision (one- or two-stage procedure). Thus, 296 cases with the removal of a prosthesis with or without the insertion of a spacer (CPT code 27,488), sepsis, or septic shock at the time of the operation were not included in the study. GNRI was then calculated for each patient using the following formula, using weight (lb) and albumin (g/L) [14–16]:

$$GNRI=\left(1.489*Albumin\right)+(41.7*\frac{Weight}{WLo})$$

WLo is the ideal weight determined by the Lorentz equation, based on gender and height (cm) [14–16]:

$${WLo}_{male}=\left(Height-100\right)+\frac{Height-150}{4}$$

$${WLo}_{female}=\left(Height-100\right)-\frac{Height-150}{2.5}$$

To not miss overweight or obese patients with malnutrition, the ratio of Weight/WLo was capped at 1 if weight exceeded WLo [14, 15].

**Fig. 1:**
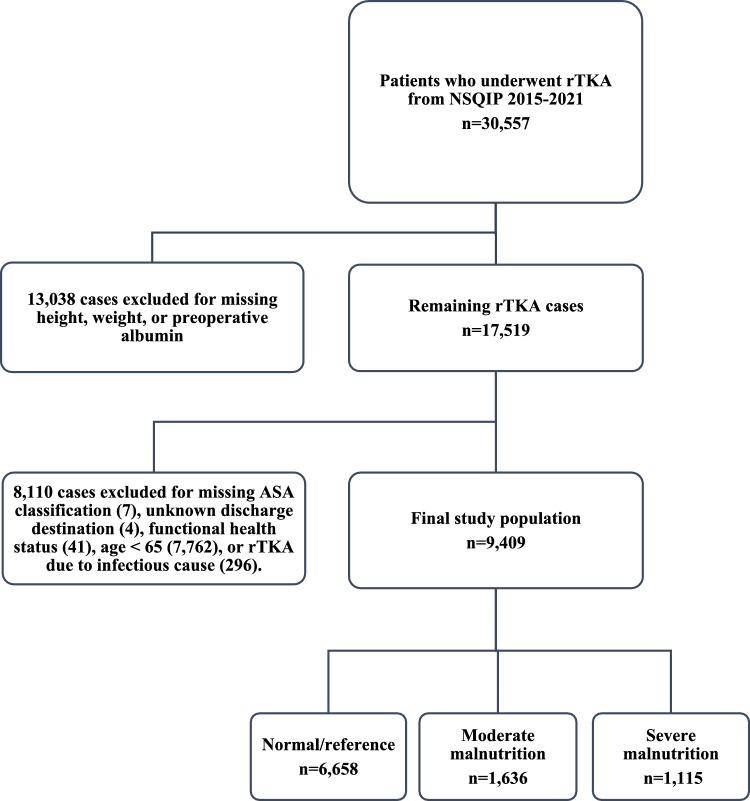
Case selection schematic. rTKA, Revision total knee arthroplasty; *NSQIP*, National surgical quality improvement program; ASA, American Society of Anesthesiologists

The remaining study population (Fig. 1) was then indexed into three cohorts based on their preoperative GNRI: normal/reference (GNRI > 98), moderate malnutrition (92 ≤ GNRI ≤ 98), and severe malnutrition (GNRI < 92). These GNRI cutoffs were chosen to stratify patients based on the severity of malnutrition, while maintaining cohort sizes of *n* > 1000. These cutoffs were have been previously validated to correlate with degree of malnutrition based on preexisting research on GNRI in TJA [14].

Variables collected in this study included patient demographics, comorbidities, surgical characteristics, and 30-day postoperative complication data. Patient demographics included gender, body mass index (BMI), age, tobacco use, functional status, ASA classification, and preoperative steroid use. Steroid use status was defined as patients who routinely used immunosuppressants or corticosteroids within 30-day pre-procedure. Tobacco use was defined as cigarette use at any point within the past year before the procedure. Preoperative comorbidities included congestive heart failure (CHF), diabetes, hypertension, severe chronic obstructive pulmonary disease (COPD), bleeding disorders, and disseminated cancer. 30-day complications included the following: sepsis, septic shock, pneumonia, unplanned reintubation, urinary tract infection (UTI), cardiac arrest or myocardial infarction (MI), stroke, blood transfusions, deep vein thrombosis (DVT), pulmonary embolism (PE), on ventilator > 48 h, surgical site infection (SSI), wound dehiscence, acute renal failure, *Clostridioides difficile* (*C. diff*) infection, non-home discharge, readmission, unplanned reoperation, length of stay (LOS) > 2 days, mortality.

All statistical analyses were conducted using SPSS Software version 26.0 (IBM Corp., Armonk, NY, USA). Patient demographics and comorbidities were compared between cohorts using bivariate logistic regression. Multivariate logistic regression, adjusted for all significantly associated patient demographics and comorbidities for the respective cohort, was used to identify associations between preoperative GNRI and postoperative complications. Odds ratios (OR) were reported with 95% confidence intervals (CI). The level of statistical significance was set at *p* < 0.05.

## Results

Compared to the normal nutrition group, the moderate malnutrition group was statistically significant for older age groups, abnormal BMI groups, dependent functional status, ASA classification ≥ 3, tobacco use, chronic steroid use, and medical comorbidities including, diabetes, hypertension, COPD, and bleeding disorders (Table 1). Compared to the normal nutrition group, the severe malnutrition group was statistically significant for older age groups, dependent functional status, ASA classification ≥ 3, chronic steroid use, and medical comorbidities including CHF, diabetes, hypertension, COPD, bleeding disorders, and disseminated cancer.

**Table 1:** Patient demographics and comorbidities for patients with preoperative normal GNRI, moderate malnutrition, and severe malnutrition

	Normal (GNRI > 98)	Moderate malnutrition (92 ≤ GNRI ≤ 98)		Severe malnutrition (GNRI < 92)	
	Number (%)	Number (%)	*p* value	Number (%)	*p* value
Overall	6658 (100.0)	1636 (100.0)		1115 (100.0)	
Sex			0.243		0.747
Female	3850 (57.8)	972 (59.4)		639 (57.3)	
Male	2808 (42.2)	664 (40.6)		476 (42.7)	
Age			** < 0.001**		** < 0.001**
65–74	4481 (67.3)	955 (58.4)		554 (49.7)	
75–84	1907 (28.6)	557 (34.0)		402 (36.1)	
≥ 85	270 (4.1)	124 (7.6)		159 (14.3)	
BMI (kg/m^2)			** < 0.001**		0.413
< 18.5	19 (0.3)	12 (0.7)		7 (0.6)	
18.5–29.9	2569 (38.6)	551 (33.7)		456 (40.9)	
30–34.9	1979 (29.7)	452 (27.6)		285 (25.6)	
35–39.9	1288 (19.3)	355 (21.7)		185 (16.6)	
≥ 40	803 (12.1)	266 (16.3)		182 (16.3)	
Functional status prior to surgery			** < 0.001**		** < 0.001**
Dependent	191 (2.9)	128 (7.8)		156 (14.0)	
Independent	6467 (97.1)	1508 (92.2)		959 (86.0)	
ASA classification			** < 0.001**		** < 0.001**
≤ 2	2271 (34.1)	387 (23.7)		131 (11.7)	
≥ 3	4387 (65.9)	1249 (76.3)		984 (88.3)	
Tobacco use			**0.017**		0.300
No	6364 (95.6)	1541 (94.2)		1,058 (94.9)	
Yes	294 (4.4)	95 (5.8)		57 (5.1)	
Steroid use			** < 0.001**		** < 0.001**
No	6357 (95.5)	1526 (93.3)		1m015 (91.0)	
Yes	301 (4.5)	110 (6.7)		100 (9.0)	
Comorbidities					
CHF	89 (1.3)	47 (2.9)	** < 0.001**	75 (6.7)	** < 0.001**
Diabetes	1486 (22.3)	419 (25.6)	**0.005**	309 (27.7)	** < 0.001**
Hypertension	4979 (74.8)	1282 (78.4)	**0.003**	889 (79.7)	** < 0.001**
COPD	323 (4.9)	120 (7.3)	** < 0.001**	113 (10.1)	** < 0.001**
Bleeding disorder	253 (3.8)	107 (6.5)	** < 0.001**	150 (13.5)	** < 0.001**
Disseminated cancer	14 (0.2)	7 (0.4)	0.124	26 (2.3)	** < 0.001**
Total operation time (minutes)			0.817		0.066
0–79	1291 (19.4)	310 (18.9)		240 (21.5)	
80–128	2049 (30.8)	526 (32.2)		347 (31.1)	
≥ 129	3318 (49.8)	800 (48.9)		528 (47.4)	

Bold *p* values indicate statistical significance with *p* < 0.05

GNRI, Geriatric nutritional risk index; BMI, Body mass index; ASA, American Society of Anesthesiologists; CHF, Congestive heart failure; COPD, Chronic obstructive pulmonary disease

Compared to the normal nutrition group, the moderate malnutrition group was significantly associated with a greater likelihood of experiencing any complication, as well as individual complications including pneumonia, blood transfusions, SSI, wound dehiscence, non-home discharge, readmission, LOS > 2 days, and mortality (Table 2). Compared to the normal nutrition group, the severe malnutrition group was significantly associated with a greater likelihood of experiencing any complication, as well as individual complications including septic shock, pneumonia, unplanned reintubation, cardiac arrest or MI, stroke, blood transfusions, on ventilator > 48 h, SSI, wound dehiscence, acute renal failure, *C. diff* infection, non-home discharge, readmission, unplanned reoperation, LOS > 2 days, and mortality.

**Table 2:** Bivariate analysis of 30-day postoperative complications in patients with preoperative normal GNRI, moderate malnutrition, and severe malnutrition

	Normal (GNRI > 98)	Moderate malnutrition (92 ≤ GNRI ≤ 98)		Severe malnutrition (GNRI < 92)	
	Number (%)	Number (%)	*p* value	Number (%)	*p* value
Any complication	3,139 (47.1)	1,058 (64.7)	** < 0.001**	980 (87.9)	** < 0.001**
Sepsis	27 (0.4)	9 (0.6)	0.427	9 (0.8)	0.073
Septic shock	6 (0.1)	3 (0.2)	0.315	9 (0.8)	** < 0.001**
Pneumonia	18 (0.3)	11 (0.7)	**0.017**	24 (2.2)	** < 0.001**
Unplanned reintubation	15 (0.2)	4 (0.2)	0.884	14 (1.3)	** < 0.001**
UTI	53 (0.8)	13 (0.8)	0.995	14 (1.3)	0.128
Cardiac arrest or MI	26 (0.4)	10 (0.6)	0.228	19 (1.7)	** < 0.001**
Stroke	8 (0.1)	1 (0.1)	0.524	8 (0.7)	** < 0.001**
Blood transfusions	323 (4.9)	208 (12.7)	** < 0.001**	252 (22.6)	** < 0.001**
DVT	43 (0.6)	13 (0.8)	0.511	13 (1.2)	0.061
PE	28 (0.4)	9 (0.6)	0.482	6 (0.5)	0.583
On ventilator > 48 h	4 (0.1)	3 (0.2)	0.144	11 (1.0)	** < 0.001**
SSI	162 (2.4)	76 (4.6)	** < 0.001**	78 (7.0)	** < 0.001**
Wound dehiscence	47 (0.7)	4 (0.2)	**0.041**	21 (1.9)	** < 0.001**
Acute renal failure	5 (0.1)	1 (0.1)	0.851	6 (0.5)	** < 0.001**
*Clostridioides difficile* infection	13 (0.2)	5 (0.3)	0.394	8 (0.7)	** < 0.001**
Non-home discharge	1,392 (20.9)	606 (37.0)	** < 0.001**	585 (52.5)	** < 0.001**
Readmission	341 (5.1)	125 (7.6)	** < 0.001**	124 (11.1)	** < 0.001**
Unplanned reoperation	214 (3.2)	54 (3.3)	0.859	57 (5.1)	**0.002**
Length of stay > 2 days	2,695 (40.5)	965 (59.0)	** < 0.001**	925 (83.0)	** < 0.001**
Periprosthetic fracture	24 (0.4)	6 (0.4)	0.97	4 (0.4)	0.993
Mortality	11 (0.2)	12 (0.7)	** < 0.001**	24 (2.2)	** < 0.001**

Bold *p* values indicate statistical significance with *p* < 0.05

GNRI, Geriatric nutritional risk index; UTI, Urinary tract infection; MI, Myocardial infarction; DVT, Deep vein thrombosis; PE, Pulmonary embolism; SSI, Surgical site infection

Adjust multivariate regression analysis controlling for all significant patient demographic and comorbidity factors (Table 3) revealed that compared to the normal nutrition group, the moderate malnutrition group was independently significantly associated with a greater likelihood of experiencing any complications, as well as individual complications including blood transfusions, SSI, non-home discharge, readmission, LOS > 2 days, and mortality. Compared to the normal nutritional group, the severe malnutrition group was independently significantly associated with a greater likelihood of experiencing any complication, as well as individual complications including septic shock, pneumonia, unplanned reintubation, cardiac arrest or MI, stroke, blood transfusions, on ventilator > 48 h, SSI, wound dehiscence, acute renal failure, non-home discharge, readmission, unplanned reoperation, LOS > 2 days, and mortality.

**Table 3:** Multivariate analysis of 30-day postoperative complications in patients with preoperative normal GNRI, moderate malnutrition, and severe malnutrition

	Moderate malnutrition (92 ≤ GNRI ≤ 98)	Severe malnutrition (GNRI < 92)
	OR, *p* value (95% CI)	OR, *p* value (95% CI)
Any complication	1.74, < **0.001** (1.55–1.95)	5.92, < **0.001** (4.89–7.17)
Septic shock	–	7.62, < **0.001** (2.57–22.61)
Pneumonia	1.69, 0.184 (0.78–3.67)	4.93, < **0.001** (2.53–9.62)
Unplanned reintubation	–	3.93, < **0.001** (1.79–8.61)
Cardiac arrest or MI	–	3.29, < **0.001** (1.73–6.27)
Stroke	0.51, 0.525 (0.06–4.09)	5.13, **0.003** (1.75–15.00)
Blood transfusions	2.33, < **0.001** (1.92–2.82)	3.85, < **0.001** (3.16–4.68)
On ventilator > 48 h	–	5.93, **0.002** (1.89–18.59)
SSI	1.74, < **0.001** (1.31–2.32)	2.61, < **0.001** (1.93–3.52)
Wound dehiscence	–	2.39, **0.002** (1.37–4.16)
Acute renal failure	–	4.84, **0.021** (1.27–18.53)
*Clostridioides difficile* infection	–	2.08, 0.149 (0.77–5.64)
Non-home discharge	1.82, < **0.001** (1.61–2.06)	2.87, < **0.001** (2.49–3.32)
Readmission	1.32, **0.011** (1.07–1.65)	1.89, < **0.001** (1.49–2.39)
Unplanned reoperation	–	1.54, **0.008** (1.12–2.12)
Length of stay > 2 days	1.83, < **0.001** (1.63–2.04)	5.45, < **0.001** (4.60–6.46)
Mortality	2.83, **0.016** (1.22–6.60)	6.95, < **0.001** (3.23–14.93)

Dashes represent associations not significant in bivariate analysis and were not included in multivariate analysis. Bold *p* values indicate statistical significance with *p* < 0.05

GNRI, Geriatric nutritional risk index; OR, Odds ratio; CI, Confidence interval; MI, Myocardial infarction; SSI, Surgical site infection

In general, compared to the normal nutrition group, severe malnutrition was independently significantly associated with a greater number of complications than moderate malnutrition. Moreover, for complications independently significantly associated with both moderate and severe malnutrition, severe malnutrition was generally found to have stronger associations: any complication (OR 1.74 in moderate malnutrition vs. 5.92 in severe malnutrition), blood transfusions (OR 2.33 vs. 3.85), SSI (OR 1.74 vs. 2.61), non-home discharge (OR 1.82 vs. 2.87), readmission (OR 1.32 vs. 1.89), LOS > 2 days (OR 1.83 vs. 5.45), and mortality (OR 2.83 vs. 6.95).

## Discussion

In this study, we found that compared to normal nutrition, moderate malnutrition was independently significantly associated with a greater likelihood of experiencing any complication, blood transfusions, SSI, non-home discharge, readmission, LOS > 2 days, and mortality. Severe malnutrition was independently significantly associated with a greater likelihood of experiencing any complication, septic shock, pneumonia, unplanned reintubation, cardiac arrest or myocardial infarction, stroke, blood transfusions, on ventilator > 48 h, SSI, wound dehiscence, acute renal failure, non-home discharge, readmission, unplanned reoperation, LOS > 2 days, and mortality. Severe malnutrition was independently significantly associated with a greater number of complications and had a stronger association with complications compared to moderate malnutrition.

Malnutrition was recently defined by the European Society of Clinical Nutrition and Metabolism as “BMI < 18.5 kg/m^2^, or an unintentional weight loss > 10% of initial body weight with BMI < 20 kg/m^2^ if < 70 years of age or BMI < 22 kg/m^2^ if older than 70 years, or fat-free mass index < 15 and 17 kg/m^2^ in women and men, respectively” [17]. While this definition has been widely used, there exist critics who advocate for the consideration of measures reflecting bodily function such as inflammation [18]. It has been well established that malnutrition downregulates the immune response by suppressing immunologic functions such as lymphocyte production and antibody secretion [19–21]. These processes may be especially detrimental in postoperative patients who need a robust immune response to repair wounds, prevent catabolic states, and fight off infections [22].

Our analysis found that both moderate and severe malnutrition were commonly significantly associated with an older demographic, dependent functional status, ASA classification ≥ 3, steroid use, CHF, diabetes, hypertension, COPD, and bleeding disorder. One study investigating the albumin-to-fibrinogen ratio as a proxy for malnutrition also found that diabetes and an ASA classification ≥ 3 were significantly associated with malnourished patients [23]. Another study reviewing the relationship between hypoalbuminemia and TJA found that malnourished patients had significant associations with dependent functional status, steroid use, tobacco use, and multiple comorbidities [24]. Moreover, CHF, bleeding disorders, and metastatic cancer have been documented as significantly associated demographics in malnourished patients who receive TJA [25]. Thus, our findings support preexisting literature showing that malnourished patients have a greater number of comorbidities compared to patients with normal nutrition.

We found both moderate and severe malnutrition to be significantly associated with an increased likelihood of experiencing any postoperative complication. The moderate malnutrition group was independently significantly associated with blood transfusions, SSI, non-home discharge, readmission, LOS > 2 days, and mortality, while the severe malnutrition group was independently significantly associated with septic shock, pneumonia, unplanned reintubation, cardiac arrest or MI, stroke, blood transfusions, on ventilator > 48 h, SSI, wound dehiscence, acute renal failure, non-home discharge, readmission, unplanned reoperation, LOS > 2 days, and mortality. Our findings support existing literature showing that malnutrition is linked to infections and wound complications following rTKA [10, 26]. Our findings also concur with the findings from a study of 531 rTJA patients that found higher 90-day complication and re-revision rates in patients with more severe malnutrition scores based on GNRI [27]. Furthermore, our results demonstrate that similar to malnourished patients who undergo TJA, malnourished patients who undergo rTKA are more likely to experience poorer outcomes postoperatively [11, 26, 28, 29].

As the average age of patients who undergo rTHA increases [4], methods for quantifying nutritional status must be sufficiently robust to differentiate between normal changes with age versus changes due to poor nutrition. GNRI was developed to quantify the risk of malnutrition in older adults as an alternative to hypoalbuminemia and BMI, which have been criticized for their one-dimensionality and inability to consider the systemic processes related to malnutrition [18]. In our study, only 0.7 and 0.6% of moderately and severely malnourished patients, respectively, had BMI < 18.5, showing that the utility of BMI in determining nutrition status is limited in isolation. However, GNRI combines features of body weight and serum albumin, using body weight to modulate the degree of albumin discrepancy required for malnourished classification. That is, patients with ideal body weight require a greater albumin abnormality to be considered malnourished based on GNRI compared to patients with lower than ideal body weight.

While other malnutrition indices such as the mini nutritional assessment (MNA) exist, GNRI has proved to have the most clinical utility, demonstrating better sensitivity in predicting three- and six-month mortality rates as well as better specificity and diagnostic power compared to MNA [2, 30, 31]. Furthermore, GNRI is a simple and efficient means of diagnosing malnutrition—only requiring height, weight, and albumin levels—and has the added benefit of not requiring a caregiver to be present [31]. For these reasons, the incorporation of GNRI as an adjuvant screening tool for malnutrition in geriatric patients undergoing rTKA should be considered.

There exist limitations in our study due to the characteristics of the NSQIP database. We were limited to short-term, 30-day postoperative outcomes, limiting our ability to draw conclusions regarding GNRI as a predictor of long-term adverse outcomes. Additionally, a substantial portion of cases were excluded due to missing albumin or weight values. Excluded patients likely received less extensive preoperative laboratory testing, may be inherently healthier than the subset of patients included in this study, and may therefore introduce some degree of selection bias. Additionally, aseptic rTKA cases were selected by excluding cases for which patients had a concurrent diagnosis of sepsis or septic shock. However, there may exist patients with PJI secondary to a local infection without diagnosis of sepsis or septic shock, limiting our ability to fully exclude PJI. Next, while there was a significant difference of mortality identified in the moderate and severe malnutrition groups, they represent a low percentage (0.7 and 2.2% vs. 0.2% in the normal group). It should be noted that the significant difference in mortality may not be reliable given low absolute occurrences in both groups. Furthermore, the database does not report information related to management, including pre- or postoperative nutritional supplementation that holds the ability to impact outcomes.

Our study contributes to the current findings of malnutrition in orthopedic surgeries, focusing on the growing population of older adults undergoing rTKA to better understand how to improve patient perioperative and postoperative treatment plans based on their risk factors.

## Conclusion

In geriatric patients with GNRI indicative of malnutrition, the overall rate of complication following rTKA was found to increase with increasing severity of malnutrition. Our results show that GNRI is a strong predictor of early postoperative complications for geriatric rTKA patients and support its utility as an adjunctive risk stratification tool for geriatric patients undergoing rTKA.

## Data Availability

No datasets were generated or analyzed during the current study.
